# Aged Callus Skeletal Stem/Progenitor Cells Contain an Inflammatory Osteogenic Population With Increased IRF and NF-κB Pathways and Reduced Osteogenic Potential

**DOI:** 10.3389/fmolb.2022.806528

**Published:** 2022-06-09

**Authors:** X. Lin, H. Zhang, J. Liu, C L. Wu, A. McDavid, B. F. Boyce, L. Xing

**Affiliations:** ^1^ Department of Pathology and Laboratory Medicine, Rochester, NY, United States; ^2^ Center for Musculoskeletal Research, Rochester, NY, United States; ^3^ Biostatistics and Computational Biology, University of Rochester Medical Center, Rochester, NY, United States

**Keywords:** skeletal stem/progenitor cell, fracture, aging, IRF, mesenchymal stroma cells, interferon regulatory factor, NF kappa b

## Abstract

Skeletal stem/progenitor cells (SSPCs) are critical for fracture repair by providing osteo-chondro precursors in the callus, which is impaired in aging. However, the molecular signatures of callus SSPCs during aging are not known. Herein, we performed single-cell RNA sequencing on 11,957 CD45^-^CD31^-^Ter119^-^ SSPCs isolated from young and aged mouse calluses. Combining unsupervised clustering, putative makers, and DEGs/pathway analyses, major SSPC clusters were annotated as osteogenic, proliferating, and adipogenic populations. The proliferating cluster had a differentiating potential into osteogenic and adipogenic lineages by trajectory analysis. The osteoblastic/adipogenic/proliferating potential of individual clusters was further evidenced by elevated expression of genes related to osteoblasts, adipocytes, or proliferation. The osteogenic cluster was sub-clustered into house-keeping and inflammatory osteogenic populations that were decreased and increased in aged callus, respectively. The majority of master regulators for the inflammatory osteogenic population belong to IRF and NF-κB families, which was confirmed by immunostaining, RT-qPCR, and Western blot analysis. Furthermore, cells in the inflammatory osteogenic sub-cluster had reduced osteoblast differentiation capacity. In conclusion, we identified 3 major clusters in callus SSPCs, confirming their heterogeneity and, importantly, increased IRF/NF-κB-mediated inflammatory osteogenic population with decreased osteogenic potential in aged cells.

## Introduction

Skeletal stem/progenitor cells (SSPCs) are multipotent cells with lineage-committed progeny and self-renewal capacity (Chan et al., 2015). SSPCs are critical for fracture repair by providing osteo-chondro-precursors in the callus. SSPCs are increased in fracture callus (Chan et al., 2015) while depletion of SSPCs with irradiation results in reduced fracture healing (Mitchell and Logan, 1998). Recent studies report that SSPCs exert inflammatory and senescent phenotypes, contributing to impaired fracture healing in aged mice (Josephson et al., 2019; Ambrosi et al., 2021). However, most of these studies use SSPCs from the long bone or bone marrow but not from fracture callus where the combination of acute injury and natural aging creates a unique microenvironment. Exploring the molecular signature of callus SSPCs and their potential influence on fracture healing during aging is important because unlike bone/bone marrow SSPCs that are derived from the growth plate, endosteum, and perivascular sites (Ambrosi et al., 2019), callus SSPCs are mainly derived from periosteum (Debnath et al., 2018) and are directly exposed to fracture injury-caused local environmental changes that are often rapid and drastic while bone/bone marrow SSPCs reside in a relatively homeostatic environment. In this study, we performed single-cell RNA sequencing (scRNAseq) on SSPCs that are directly isolated from young and aged mouse callus to discover callus SSPC subsets, gene profiles, and master regulators that may affect fracture healing in aging. We also validated our scRNA-seq findings with immunostaining, RT-qPCR, and Western blot analysis and explored the functional implication of increased inflammatory osteogenic cells in aged callus.

## Materials and Methods

### Animals and Tibial Fracture Procedure

Young (4-month-old, equivalent to 26-year-old in humans) and aged (21-month-old, equivalent to 62-year-old in humans) C57BL/6J mice from the National Institute on Aging were used. Mice were housed in micro-isolator technique rodent rooms. All animal procedures were approved by the University Committee on Animal Research at the University of Rochester. Open tibial fractures were performed according to the standard Operating procedure established in the Center for Musculoskeletal Research (Brown et al., 2014). In brief, an incision of 6 mm in length was made in the skin on the anterior side of the tibia after anesthesia. A sterile 27 G × 1.25-inch needle was inserted into the marrow cavity of the tibia from the proximal end, temporarily withdrawn to facilitate transection of the tibia using a scalpel at midshaft, and then reinserted to stabilize the fracture. The incision was closed with 5–0 nylon sutures. Fractures were confirmed by radiograph. Callus tissues were harvested on day 10, the time when soft callus is formed, following the fracture procedure for cell preparation.

### Preparation of Callus Skeletal Stem/Progenitor Cells and Isolation of Cells in the Individual Cluster

For the preparation of callus SSPCs for scRNAseq, two soft calluses were dissected from the fractured tibiae on day 10 of young or aged mice and pooled as a sample, cut into small pieces (<1 mm^3^), and digested in 10 ml of Accumax solution (STEMCELL, 1 h, room temperature). Cells were passed through a 35 mm-filter, and red blood cells were lysed with ammonium chloride (5 min, room temperature). Cells were resuspended in a staining medium (PBS with 2% fetal bovine serum) and stained with APC-anti-CD45 (Biolegend, clone 30-F11), FITC-anti-CD31 (eBioscience, clone 390), PerCP/Cy5.5-anti-Ter119 (Biolegend, clone TER-119) antibodies, and Dapi. Callus SSPC cells (CD45^-^CD31^-^Ter119^-^Dapi^-^) were sorted (85-micron nozzle) by FACS with a BD Aria II instrument. For isolation of cells in individual clusters for qPCR validation, callus cells from young mice on day 10 post fracture were prepared as described earlier. Cells were stained with PE/Cy7-anti-CD45 (Biolegend, clone 30-F11), FITC-anti-CD31 (eBioscience, clone 390), PerCP/Cy5.5-anti-Ter119 (Biolegend, clone TER-119), APC-anti-CXCR2 (Biolegend, clone SA044G4), PE-anti-CCR2 (Biolegend, clone SA203G11) antibodies, and Dapi.

### scRNAseq

Cells were loaded onto a chromium chip (10X Genomics) followed by encapsulation in a lipid droplet (Single Cell 3′ kit, 10X Genomics) to generate cDNA and library according to the manufacturer’s protocol. cDNA libraries were sequenced to an average of 100,000 reads per cell using Illumina Nextseq 500. scRNA-seq reads were processed with Cell Ranger v2.1, which demultiplexed cells from different samples and quantified transcript counts per putative cell; 5,648 young cells and 7,197 aged cells were sequenced.

### Quality Control and Processing of scRNA-Seq Data.

The quality control and cell cluster identification were performed using the Seurat4 R package. After filtering out low-quality cells (<1,000 unique genes, >8% mitochondrial reads) and potential doublets [>12,000 unique molecular identifiers (UMIs)], 5,123 young and 6,834 aged cells were further analyzed. Data were then preprocessed with the Seurat4.0 R package. Data were normalized based on regularized negative binomial models with the SC Transform function. The top 2,000 variable genes were identified and ranked by coefficient of variation. Dimensionality reduction of datasets was performed by the “RunPCA” function with 25 principal components (npcs = 25) at a resolution of 0.1. Find Neighbors function was used to compute the shared nearest-neighbor (SNN) for a given dataset with parameter k = 20. Clusters of the cells were identified based on SNN modularity optimization with the Find Clusters function. The “RunUMAP” function was further used to perform Uniform Manifold Approximation and Projection (UMAP) dimensional reduction. Cell clusters were visualized on reduced UMAP dimensions using the “DimPlot” function. Differentially expressed genes (DEGs) of each cluster were identified with the “FindAllMarkers” function. The top 10 DEGs with the highest average log2-fold-change were presented in a heatmap using the “DoHeatmap” function for cluster functional annotation.

### Pathway Analysis

DEGs identified with the “FindAllMarkers” function in Seurat/R and with an average log2-fold-change > 2 and *p* value < 0.05 were uploaded to Ingenuity Pathway Analysis software (Qiagen). The top 5 pathways with the highest log10-fold-change were presented and used for cluster functional annotation.

### Pseudotime Ordering and Lineage Trajectory Analysis

Monocle2/R package (Qiu et al., 2017) was used to compute and order the sequence of gene expression changes of the cells from each cluster. First, cluster 4 cells that express B cell-related genes were removed from the dataset, which was subsequently converted to a Monocle2 object using the Seurat Wrappers R package. Monocle2 objects were processed with “estimateSizeFactors,” “estimateDispersions,” “detectGenes,” and “reduceDimension” (with “DDRTree” method) functions sequentially to order the cells along a pseudotime trajectory.

### Transcription Factor Binding Motif Analysis

To obtain the upstream master regulators and transcription factor (TF) binding motifs that regulate the callus SSPCs, we used DEGs from each cluster, respectively, as input genes to the RcisTarget R package (Aibar et al., 2017) with default parameters and mm9-tss-centered-10kb-7species.mc9nr.feather as the database. For each cluster, the top TF with the corresponding binding motif was selected and visualized. The interaction map between TFs was constructed using STRING v11.0 (Szklarczyk et al., 2019).

### Ligand Receptor Analysis

To identify the ligand**–**receptor pairs among the SSPC sub-clusters (i.e., clusters 1.1, 1.2, 2, and 3), we converted the Seurat object to CellChat/R object, a publicly available database of 2,021 validated molecular interactions for Mus Musculus, for ligand**–**receptor identification (Jin et al., 2021). The ligand**–**receptor regulatory potential and downstream target genes in the receptor cells were predicted using the Nichenetr/R package (Browaeys et al., 2020). Cluster 3 was set as the sender cell while the potential target receptors and genes were predicted for clusters 1.1 and 1.2, respectively.

### Immunostaining

Tibiae were fixed in 10% formalin for 48 h at 4°C, decalcified in 10% EDTA for two weeks, processed, and embedded in optimal cutting temperature compound (Tissue-Tek). Seven-micron sagittal sections were cut and probed with antibody for CXCR2 (Invitrogen, cat# PA5-100951), followed by goat-anti-rabbit 488 (Abcam, cat# ab150081), and counterstained with Dapi. Sections were imaged with a Zeiss AxioImager motorized fluorescent microscope system with an AxioCam camera. The percentage of CXCR2+/Dapi+ total cells within the callus is quantified with ImageProPlus software.

### Osteoblast Differentiation Assay and Alkaline Phosphatase Staining

Callus pieces were prepared with Accumax digestion as described earlier and cultured in α-MEM medium containing 15% fetal bovine serum. Cells that were migrated from callus were continuously cultured to 3rd–5th passages as callus mesenchymal progenitor cells. CXCR2^low^ and CXCR2^high^ callus mesenchymal progenitor cells were purified using CXCR2 antibody (Biolegend cat#149304, clone SA044G4) by fluorescence-activated cell sorting (FACS) or magnetic beads sorting (Miltenyl cat#130-048-801). CXCR2^low^ and CXCR2^high^ cells were seeded in a 96-well plate (1 × 10^4^/well), cultured to 60% confluence, and then cultured in the osteoblast-inducing medium (α-MEM medium containing 15% fetal bovine serum, 50 μg/ml ascorbic acid, and 10 mM β-glycerophosphate) for seven days. The cells were harvested with 100% ethanol for 10 min, washed 3 times with PBS, and stained with 1-step NBT/BCIP reagent for alkaline phosphatase (ALP).

### RT-qPCR

Cells that were isolated directly from fracture callus as individual clusters or CXCR2+ or CXCR2- callus mesenchymal progenitor cells were subjected to RNA extraction. RNA was extracted in TRIzol, and cDNA was synthesized using the iSCRIPT cDNA synthesis kit (BioRad). qPCR was performed using primers in Supplementary Table S1 with iQ SYBR Green Supermix using an iCycler PCR machine (BioRad). The fold change of gene expression was first normalized to actin and then normalized to the values in cluster 1 Dapi-Ter119-CD45-CD31-CXCR2+CCR2- cell or CXCR2^low^ cells, respectively.

### Western Blot Analysis

Callus tissues were homogenized after being frozen in liquid nitrogen with a mortar and pestle. Homogenized tissues or cells were lysed in protein lysis buffer containing 1x RIPA buffer (EMD Millipore 20-188), 1 mM DTT (Sigma-Aldrich), 1 mM PMSF (Sigma-Aldrich), and 5 mM N-ethylmaleimide (Millipore Sigma 10197777001) and protease inhibitor cocktail (Millipore Sigma 04693116001). Proteins were loaded onto 15% SDS-PAGE gel and blotted with anti-IFITM1 Ab (Cell Signaling 13126, 1:500) and anti-S100A6 Ab (Cell Signaling 13162, 1:500).

### Statistical Analysis

Statistical analysis was performed using GraphPad Prism 5 software (GraphPad Software Inc., San Diego, CA, United States). Data are presented as mean ± SD. Comparisons between two groups were analyzed using a two-tailed unpaired Student’s t-test. Comparisons among 3 groups were analyzed using one-way ANOVA followed by the Tukey post-hoc test.

## Results

### Unsupervised Clustering Reveals 3 Major Clusters in Callus Skeletal Stem/Progenitor Cells

**FIGURE 1:**
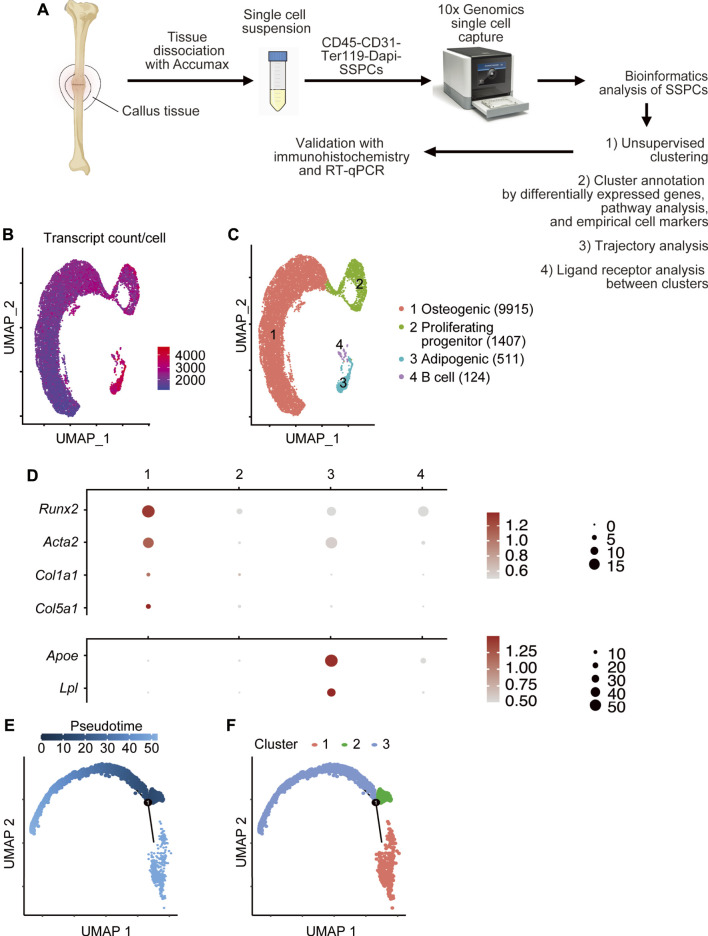
Single-cell RNA sequencing of fracture callus CD45^-^CD31^-^Ter119^-^ SSPC cells. **(A)** Isolation of callus SSPCs for scRNAseq. The tibial fracture was performed on 4-month- (young) and 21-month-old (aged) C57BL/6J male mice, and fracture callus was harvested 10 days after the procedure. A single-cell suspension was prepared by pooling two fracture calluses. CD45^-^CD31^-^Ter119^-^Dapi^-^ SSPC cells were sorted with FACS and subjected to scRNAseq. Data were analyzed using bioinformatics methods and validated by immunohistochemistry and RT-qPCR. **(B)** The number of transcripts per cell is demonstrated on UMAP. **(C)** A total of 11,957 cells were subjected to unsupervised SNN clustering using Seurat/R and resolved four major clusters. The number of cells within each cluster is indicated in the figure legend: 9,915 cells in the osteogenic cluster, 1,407 cells in the proliferating progenitor cluster, 511 cells in the adipogenic cluster, and 124 cells in the B cell cluster. **(D)** Expression of putative osteogenic and adipogenic markers of SSPCs in clusters 1–3: *Acta2* (SSPC), *Runx2* (osteogenic), *Col1a1* and *Col5a1* (osteoblast), and *Apoe* and *Lpl* (adipocyte). **(E,F)** Pseudotime analysis of cluster 1–3 with Monocle2 demonstrating that cluster2 proliferating progenitor was the earliest along the developmental tree according to pseudotime alignment and could differentiate into cluster 1 osteogenic cells and cluster 3 adipogenic cells.

To examine the SSPC populations in callus tissues during the early phase of bone fracture healing, a time when SSPCs were rapidly expanding to form the soft callus, we performed scRNA-seq analysis on purified SSPCs defined as CD45^-^CD31^-^Ter119^-^ cells (Supplementary Figure S1). This gating strategy would exclude hematopoietic lineage cells *via* CD45 negative selection, endothelial cells *via* CD31 negative selection, and erythroid lineage cells *via* Ter119 negative selection. In order to acquire a comprehensive profile of SSPCs that contributed to fracture repair, we used the triple-negative selection of CD45, CD31, and Ter119 for classically defined stromal cells (Morikawa et al., 2009; Omatsu et al., 2010; Worthley et al., 2015), compared to the more stringent CD45-Ter119-PDPN+CD146-CD73+CD164+ mouse skeletal stem cells defined by Chan et al. (2015) to acquire a comprehensive profile of stromal cells that contributed to fracture repair. A scalable droplet-based scRNA-seq platform (10X Genomics Chromium) was used to profile FACS-purified live (DAPI-negative) SSPCs from the callus of young (4-month-old) and aged (21-month-old) mice following tibial fracture (Figure 1A, Supplementary Figure S1). A total of 12,845 cells were sequenced at an average depth of 100,000 reads per cell, with 5,648 cells from young and 7,197 cells from aged mice, respectively. The two libraries were aggregated and aligned using the Cell Ranger pipeline (10X Genomics) to compensate for minor differences in library complexity. After quality control filtering to remove cells with low gene detection (<500 genes) and high mitochondrial gene content (>8%), 5,123 young cells and 6,834 aged cells were used for clustering and cell-type identification analysis of combined young and aged datasets using Seurat4.0.3/R (Satija et al., 2015). Figure 1B demonstrated that all cells in the analysis had sufficient transcript counts per cell.

Initial unsupervised clustering revealed four clusters (Figure 1C). To define these cell clusters, we first assessed the expression of putative markers for SSPCs, specifically osteoblastic markers and adipoblastic markers. *Acta2*, a gene commonly used in lineage tracing and in scRNA-seq study for SSPCs (Josephson et al., 2019; Matthews et al., 2021), and *Runx2* (Komori, 2010), the master transcription factor for osteogenesis, were expressed in cluster 1, which also expressed osteoblast markers (*Col1a1* and *Col5a1*) (Dacic et al., 2001; Lin et al., 2020) (Figure 1D) and fibroblast markers *[Pdpn* (Astarita et al., 2012)] (Supplementary Figure S2). Adipocyte markers *Apoe* and *Lpl* (Moseti et al., 2016) were expressed in cluster 3 (Figure 1D). No chondrocyte-related genes (*Sox9* and *Col2a1*) (Sive et al., 2002) (Figure 1D) were detected. Cluster 2 expressed proliferative markers including *Mki67*, *Top2a,* and histones. Cluster 4 expressed genes related to B cells (*Cd79* and *Cd19*) with upregulated B cell pathways (Supplementary Figure S3) (Adams et al., 2009). Since B cells are CD45^+^ hematopoietic cells despite our CD45^-^CD31^-^Ter119^-^ gating strategy for FACS sorting, 124 cells in cluster 4 were likely due to contamination and thereby were removed from subsequent bioinformatics analysis.

To better understand the differentiation hierarchy of SSPC clusters, we performed trajectory analysis to re-order cells along a pseudotime with Monocle2 and found consistent results that cluster 2 was the earliest along the pseudotime trajectory (Figure 1E) and could be labeled as the root cells that could differentiate into cluster 1 and cluster 3 populations (Figure 1F).

To further characterize purified CD45^-^CD31^-^Ter119^-^ cells, we assessed the expression of putative markers for hematopoietic populations (CD45^+^) and endothelial cells (CD31^+^). The expression of *Cd3g*, *Trac* (T cell), *Ighg1* (plasma cell), *Tpsab1* (mast cell), *Krt6a* (epithelial cell), and *Emcn* and *Vwf* (endothelial cells) was low or undetectable while expression of *Cd14* (monocyte), *Adgre* (macrophage), and *Fut4* (neutrophil) was low to moderate (Supplementary Figure S2).

### Differentially Expressed Genes and Pathway Analysis Further Define the Osteogenic, Proliferating-Progenitor, and Adipogenic Clusters

**FIGURE 2:**
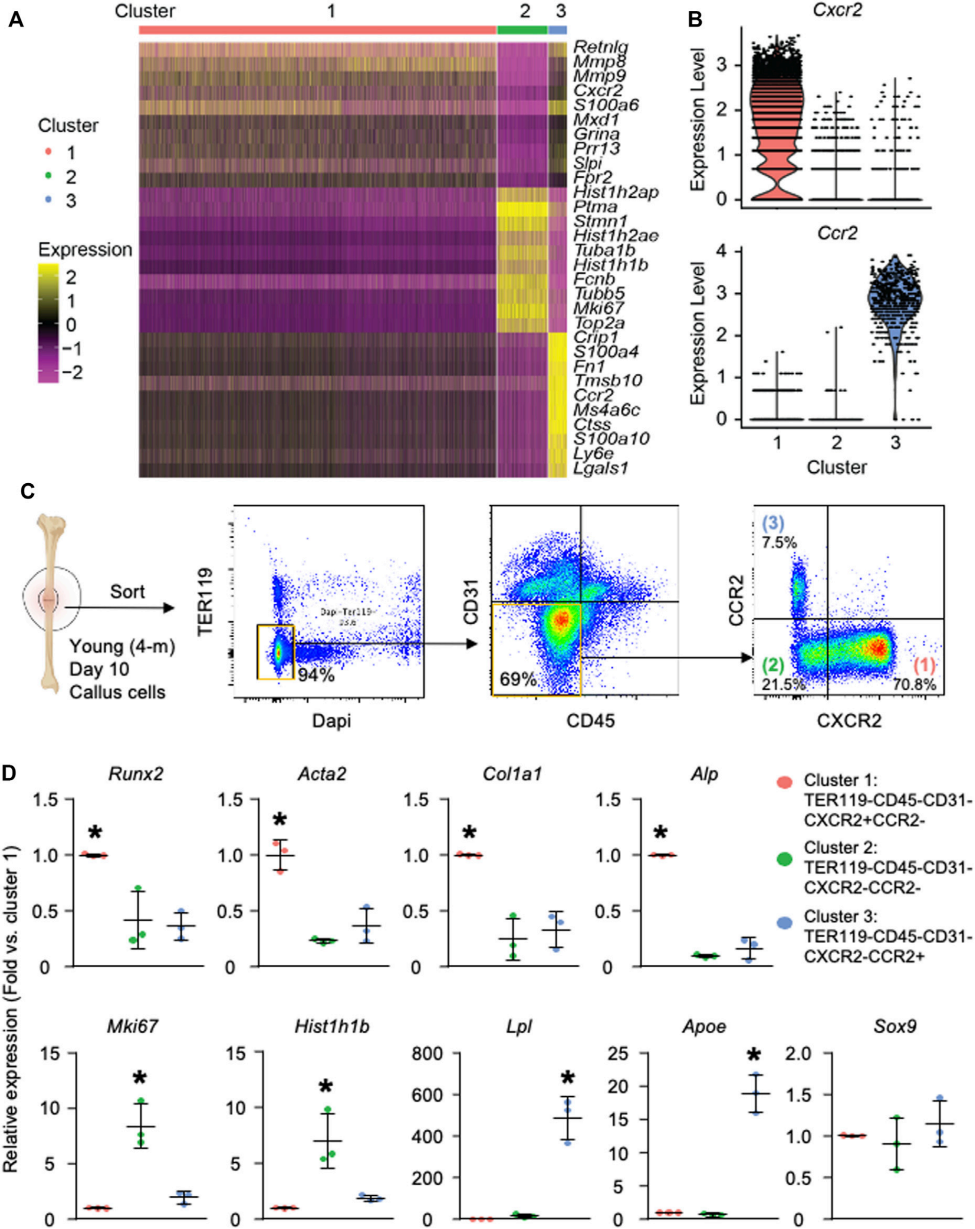
Callus SSPCs are composed of osteogenic, proliferating, and adipogenic clusters. Analysis was performed on clusters 1–3. Cluster 4 was excluded from further analysis due to possible contamination of B cells during cell harvest, which is described in Supplementary Figure S2. **(A)** Heatmap of the top 10 DEGs in clusters 1–3 showing cluster 1 expressed inflammatory and matrix related genes, cluster 2 expressed proliferating genes, and cluster 3 expressed a miscellany of inflammatory, stem cell-related, and oncogenes. **(B)** Violin plot of the expression level of surface markers, Cxcr2 and Ccr2, within clusters 1–3. **(C)** Isolation of clusters 1–3 using FACS sorting: Cluster 1: Dapi-TER119-CD45-CD31-CXCR2+CCR2-; Cluster 2: Dapi-TER119-CD45-CD31-CXCR2-CCR2-; Cluster 3: Dapi-TER119-CD45-CD31-CXCR2-CCR2+. **(D)** RT-qPCR of cluster 1–3 cells to assess their osteogenic, proliferative, and adipogenic capacity. Data represent mean ± SD. One-way ANOVA followed by Tukey post-hoc. *p < 0.05 for significant difference from any other clusters.

Heatmap of top 10 DEGs in each cluster (Figure 2A) showed that cluster 1 expressed inflammatory [*Retnlg* (Nagaev et al., 2006) and *Cxcr2* (Kawagoe et al., 2020)], and matrix genes [*Mmp8* (Henle et al., 2005) and *Mmp9* (Colnot et al., 2003)]; cluster 2 expressed genes associated with cell proliferation [*Mki67* (Sun and Kaufman, 2018) and *Tuba1b* (Lu et al., 2013)], chromosome regulation [*Top2a* (Nielsen et al., 2020)], and histone modification (*Hist1h2ap* and *Hist1h1b*) (Marzluff et al., 2002); and cluster 3 expressed a miscellany of genes, including oncogene *Crip1* (Ludyga et al., 2013), matrix-related *Fn1* (Klavert and van der Eerden, 2021), inflammatory *S100a4* (Ambartsumian et al., 2019), and stem-cell marker *Sca1* homolog *Ly6e* (Upadhyay, 2019). Pathway analysis revealed that cluster 1 had multiple upregulated pathways that mediate cell adhesion, an important cellular process of inflammation (Supplementary Figure S4). Cluster 2 mainly had upregulated pathways related to cell division and cell cycle regulation (Supplementary Figure S5). The oxidative phosphorylation pathway and eIF4/p70S6K pathway that are important for adipocyte differentiation (Le Bacquer et al., 2007) were elevated in cluster 3 (Supplementary Figure S6). Combining unsupervised clustering, conventional makers, and DEGs/pathway analyses, we annotated clusters 1–3 as the osteogenic population, proliferating-progenitors, and adipogenic population, respectively (Figure 1C).

To validate the three major subpopulations of SSPC and their osteogenic/adipogenic/chondrogenic stemness experimentally, we first searched for surface proteins among the DEGs identified in Figure 2A and selected CXCR2 from cluster 1 and CCR2 from cluster 3 for our sorting strategy (Figure 2B). We sorted CD45^-^CD31^-^Ter119^-^CXCR2^+^CCR2^-^ cluster 1, CD45^-^CD31^-^Ter119^-^CXCR2^-^CCR2^-^ cluster 2, and CD45^-^CD31^-^Ter119^-^CXCR2^-^CCR2^+^ cluster 3 cells from callus cells directly isolated from the callus of wild type mice at 10 days post-fracture (Figures 2B, C). We performed RT-qPCR on them to examine the expression of genes related to osteogenic/adipogenic/chondrogenic stemness and cell proliferation. We found that cluster 1 cells expressed the highest level of osteoblastic genes (*Alp*, *Runx2, Col1a1*, and *Acta2*); Cluster 2 cells expressed the highest level of proliferative genes (*Mki67* and *Hist1h1b*); and cluster 3 cells expressed the highest level of adipogenic genes (*Lpl* and *Apoe*). Similar to low levels of genes associated with chondrogenesis, cells in all 3 clusters expressed low levels of chondrogenic genes (*Sox9*) (Figure 2D).

### Aged Callus Skeletal Stem/Progenitor Cells Contain an Inflammatory-Osteogenic Subset With Increased Genes in Interferon Response Factor Pathways

**FIGURE 3:**
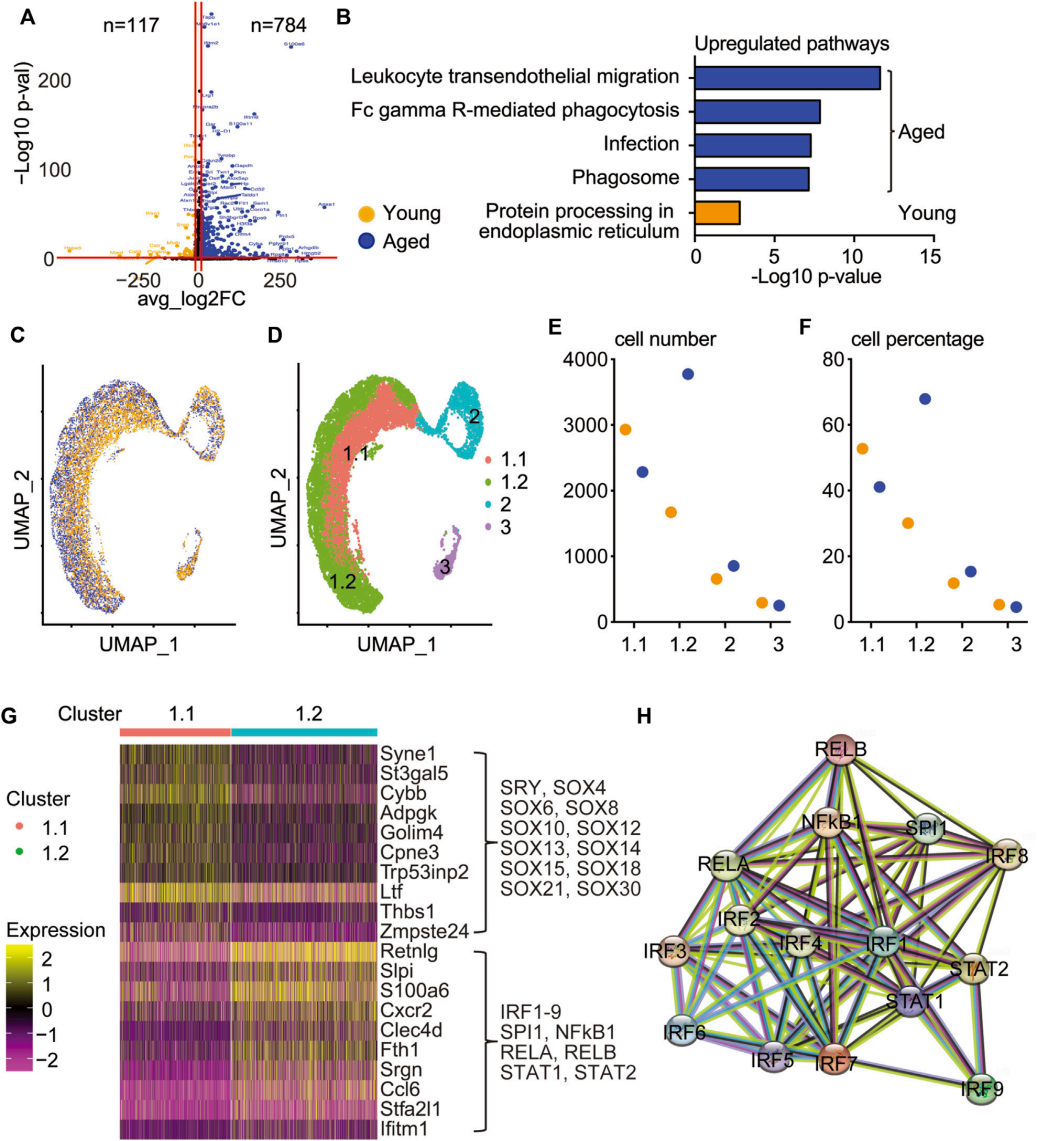
Aged callus SSPCs contain an inflammatory osteogenic sub-cluster with upregulated IRF pathway. Bioinformatics analysis of clusters 1–3 comparing young and aged SSPCs. **(A)** Upregulated genes in young and aged SSPCs were demonstrated on the volcano plot, with each dot representing an individual gene. 117 and 784 genes with a log2FC greater than 2 and a *p*-value less than 0.05 in young and aged samples were colored in orange and blue, respectively. **(B)** Upregulated pathways in young and aged SSPCs were identified using the 117 and 784 upregulated genes in young and aged callus. Only one upregulated pathway was identified in young callus SSPCs, while the top pathways upregulated in aged callus SSPCs were related to inflammation. **(C)** UMAP demonstrated that the distribution of young and aged SSPCs was separated in cluster 1 osteogenic cluster. **(D)** Cluster 1 osteogenic cells were sub-clustered into 1.1 and 1.2 according to the separation of young and aged. **(E,F)** The cell number and percentage of cluster 1.1 were higher in young and vice versa for cluster 1.2 and were comparable in clusters 2 and 3. **(G)** Heatmap showing top 10 DEGs of clusters 1.1 and 1.2 revealing increased genes related to house-keeping and inflammation, respectively, which were driven by SOX family and IRF family transcription factors, respectively, identified by RcisTarget/R analysis. **(H)** The interaction map between master regulators of cluster 1.2.

To explore the difference in molecular signature between young and aged callus SSPCs, we identified 117 and 784 upregulated DEGs in young and aged SSPCs, respectively (Figure 3A). Pathway analysis revealed that aged cells had increased inflammatory pathways while young cells had increased protein processing pathways (Figure 3B). The distribution of young and aged cells on UMAP revealed a clear separation of young and aged cells within cluster 1 osteogenic population (Figure 3C), which was further sub-clustered into 1.1 and 1.2 subsets (Figure 3D). Interestingly, the cell number and percentage in cluster 1.1 decreased while cells in cluster 1.2 increased in aged SSPCs. Cell numbers in cluster 2 and cluster 3 did not exhibit a clear difference between young and aged cells (Figures 3E, F). Heatmap showed that the DEGs of cluster 1.1 were genes related to house-keeping function, including metabolic-related [*Adpgk* (Imle et al., 2019) *and Cybb* (Frazão et al., 2015)], cytoskeletal-related [*Syne1* (Rajgor and Shanahan, 2013) and *Golim4* (Lu et al., 2017), *Ltf* (Cornish et al., 2004; Naot et al., 2005)], and matrix-related [*Zmpste24* (Bergo et al., 2002) *and Thbs1* (Yamashiro et al., 2020)] genes. In contrast, the DEGs of cluster 1.2 were enriched in inflammatory genes [*S100a6* (Xia et al., 2017)*, Cxcr2* (Xia et al., 2016)*,* and *Ifitm1* (Liao et al., 2019)] (Figure 3G). To further characterize the DEGs in these two sub-clusters, we performed transcription factor analysis to infer their upstream regulators by the RcisTarget R package (Aibar et al., 2017). The master regulators for cluster 1.1 were the Sox family transcription factors (Figures 3F, G) and for cluster 1.2 belonged to the IRF-STAT and NF-κB pathways (Figures 3F, G). Since Sox family transcription factors regulate multiple cellular functions while IRF-STAT and NF-κB pathways are mainly involved in inflammation, we further sub-clustered the osteogenic cluster in Figure 1C and named them as cluster 1.1 house-keeping osteogenic progenitors and cluster 1.2 inflammatory osteogenic progenitors.

To explore the interactions among the SSPC clusters, we performed a ligand–receptor analysis using the CellChat/R package (Jin et al., 2021). CellChat analysis revealed that cluster 3 adipogenic progenitors expressed the most outgoing signals (ligands), whereas cluster 1.1 house-keeping osteogenic and cluster 1.2 inflammatory osteogenic progenitors were the major receivers of incoming signals (Supplementary Figure S7). To identify the potential downstream signals activated by the cluster 3 outgoing ligands to cluster 1.1 and cluster 1.2, we utilized the Nichenetr/R package, a computational method to infer the intracellular communications by linking ligands to target genes (Browaeys et al., 2020). Among the inferred target genes of cluster 1.1 that did not overlap with cluster 1.2, *Irf2bp2* has been identified as a repressor for IRF2 signaling (Ramalho-Oliveira et al., 2019). In contrast, among the five target genes of cluster 1.2 that were not inferred in cluster 1.1, *Cebpb* (Pal et al., 2009), *Acvrl1* (Verma et al., 2016), *Anxa1* (Yap et al., 2020), and *Upp1* (Yan et al., 2016) have been reported to be related to IRF signaling and inflammation in various disease models, supporting the argument that cluster 1.2 has the inflammatory osteogenic progenitors mediated by IRF signaling (Supplementary Figure S8).

### Increased Inflammatory CXCR2^high^ Cells in the Callus of Aged Mice With Elevated Expression of Interferon Response Factor and NF-κB Response Genes and Reduced Osteogenic Potential

**FIGURE 4:**
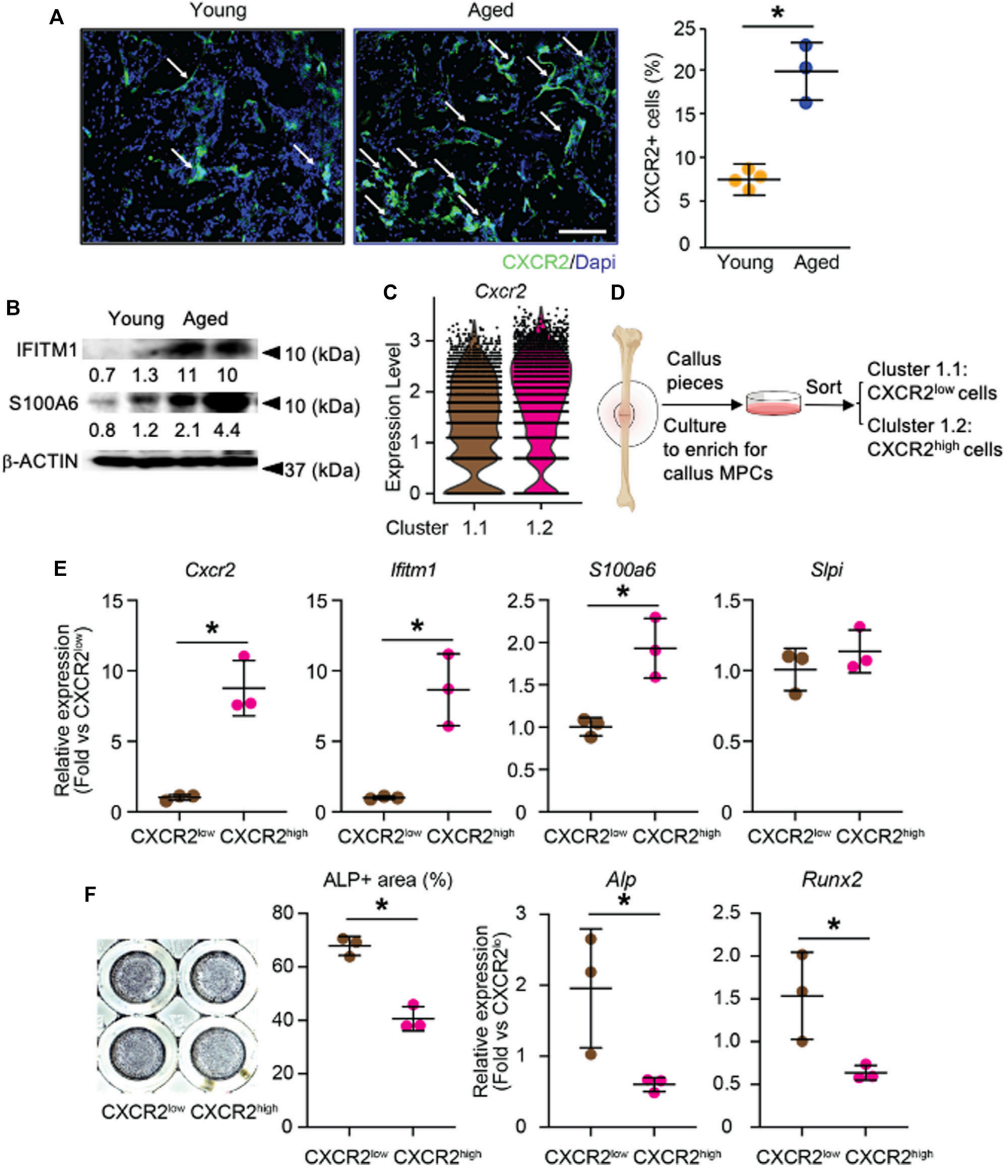
Validation of increased CXCR2^high^ cells in the callus of aged mice with increased IRF and NF-κB pathways and reduced osteogenic potential. The tibial fracture was performed on 4-month- (young) and 21-month-old (aged) C57BL/6J male mice and fracture callus was harvested 10 days after the procedure. **(A)** Immunostaining with anti-CXCR2 antibody in callus sections for CXCR2+ cells. Images show numerous CXCR2+ cells (arrows) localize on the surface of woven bone. The percentage of CXCR2+ cells in the callus of young and aged mice was quantified by Image J software. *n* = 4 mice/young and 3 mice/aged. Data represent mean ± SD. Unpaired two-tailed t-test. *p < 0.05. WB: Woven bone. Scale bar = 1 mm. **(B)** Western blot to assess IRF-response protein IFITM1 and NF-κB-response protein S100A6 in young and aged callus tissues. **(C)** Violin plot of the expression level of Cxcr2 in cluster 1.1 and cluster 1.2. **(D)** CXCR2^low^ and CXCR2^high^ cells were isolated from callus-derived mesenchymal progenitor culture. **(E)** The expression levels of target genes for IRF and NF-κB were measured by RT-qPCR. *n* = 3 mice/group. Data represent mean ± SD. Unpaired two-tailed t-test. *p < 0.05. **(F)** CXCR2^low^ and CXCR2^high^ cells isolated from callus-derived mesenchymal progenitor culture were cultured in osteoblast inducing medium for 7 days and stained for ALP. The percentage area of ALP+ was measured in Image J software. Expression level of *Alp* and *Runx2* was measured with RT-qPCR. Data represent mean ± SD. Unpaired two-tailed *t*-test. *p < 0.05.

To validate our finding that inflammatory osteogenic progenitors are increased in aging (Figures 3C, D), we performed immunostaining for CXCR2, one of the top DEGs in this sub-cluster (Figure 3G), on young and aged fracture callus tissue sections. Immunohistochemistry detected numerous CXCR2+ cells in the callus and they were mainly localized on the surface of woven bones in both young and aged mice. The percentage of CXCR2+ cells was significantly increased in aged callus (Figure 4A). We then assessed the protein levels of IRF-response gene, interferon-induced transmembrane protein 1, *Ifitm1* (Ogony et al., 2016), and NFκB-response gene, *S100a6* (Joo et al., 2003) in young and aged callus, which were among the top DEGs of cluster 1.2 that was predominant in aged callus (Figure 3G). Elevated levels of IFITM1 and S100A6 were detected in aged samples (Figure 4B). To experimentally validate the inflammatory and functional phenotypes of cluster 1.1 and 1.2 cells, we cultured callus pieces to generate callus-derived mesenchymal progenitor cells as we recently described (Liu et al., 2022) and used surface marker CXCR2 to separate the two sub-clusters in cluster 1. Because the expression of *Cxcr2* was higher in cluster 1.2 compared to cluster 1.1 (Figure 3G, Figure 4C), we isolated CXCR2^low^ cells as cluster 1.1 cells and CXCR2^high^ cells as cluster 1.2 cells (Figure 4C). The expression of *Cxcr2* was about 10-fold higher in CXCR2^high^ cells as a positive control of CXCR2^high^ cell purification. Consistent with the increased corresponding protein levels in aged callus tissues, fitm1 and S100a6 mRNA levels were ∼9-fold and ∼2-fold higher in CXCR2^high^ cells than in CXCR2^low^ cells, respectively (Figure 4D). However, the expression of *Slpi*, an IRF-response gene, was comparable between CXCR2^low^ and CXCR2^high^ cells (Figure 4E). These data demonstrated that aged mice had increased callus CXCR2^high^ cells that expressed high levels of IRF and NF-κB response genes. Finally, we assessed the osteogenic capacity of CXCR2^high^ cells to explore their functional implication and found that CXCR2^high^ cells had less ALP+ staining area and decreased *Alp* and *Runx2* mRNA expression levels (Figure 4F). This indicates that the reduced osteogenic capacity in inflammatory-osteogenic SSPCs may play a role in decreased fracture healing during aging.

## Discussion

Our scRNA-seq study identified three major clusters in callus SSPCs, e.g., osteogenic, proliferating precursors, and adipogenic clusters, confirming the heterogeneity of SSPCs. More importantly, we found that the osteogenic cluster can be further divided into house-keeping and inflammatory sub-clusters, based on significantly differential gene expression profile between young and aged cells, which may indicate the different osteogenesis between young and aged mice during bone fracture healing. Compared to the young osteogenic cluster, the aged osteogenic cluster is composed of fewer house-keeping and more inflammatory cells. Increased *Cxcr2* and several other inflammatory genes were detected in the aged inflammatory osteogenic population (cluster 1.2 in Figure 3), which was confirmed by immunostaining of CXCR2 on callus sections and by qPCR of IRF and NF-κB target genes in CXCR2^high^ cells, which had decreased osteogenic capacity. Based on these findings, we propose a model to illustrate callus SSPC subsets and their potential roles in fracture healing in aging. In fracture callus, SSPCs can be divided into three clusters: osteogenic, proliferating, and adipogenic populations. The major difference between young and aged mice is the osteogenic population because it can be sub-clustered into the house-keeping osteogenic and inflammatory osteogenic populations. In young mice, the house-keeping osteogenic precursors promote fracture repair by providing metabolic and matrix synthetic functions as well as limiting the development of inflammatory osteogenic population *via* the production of IRF-inhibiting signals. In aged mice, the house-keeping osteogenic population decreased while the inflammatory osteogenic population increased. These inflammatory osteogenic cells express high levels of chemokine receptor *Cxcr2* and other target genes of IRF and NF-κB pathways, which is confirmed in CXCR2^high^ callus cells. Specific targeting of the inflammatory osteogenic population may present a new therapeutic approach for fracture healing in the elderly.

Although a plethora of transcriptomic analyses have been performed on the fracture repair model, our study still provides further insights into the heterogeneity of non-hematopoietic SSPCs directly isolated from young and aged fracture callus tissue. Most importantly, we identified a unique CXCR2 expressing inflammatory osteogenic population and demonstrated that aging increased this inflammatory osteogenic population post-fracture. Our result is consistent with a recent report, in which microarray on callus tissue in 5-month-old and 25-month-old mice revealed increased inflammatory response including increased expression of *Ccl* and *Cxcl* family chemokines in aged mice (Hebb et al., 2018). scRNAseq on callus Ter119- cells of 24-month-old mice revealed that mice treated with vehicle had a prominent enrichment of cells expressing myeloid genes while mice treated with a combination of BMP2 and low dose anti-CSF1 had increased cells expressing SSPC genes and enhanced fracture healing that is comparable to the young mouse level and increasing cells expressing SSPC genes. In their study, the authors proposed that myeloid cells may inhibit SSPCs on fracture healing by creating an inflammatory-degenerative niche in the callus (Ambrosi et al., 2021). Our study indicates that in addition to myeloid cells, osteogenic cells may also function as inflammatory cells.

We found that Cxcr2 is a top DEG for the inflammatory osteogenic population that is increased in aged callus and CXCR2^high^ cells have elevated expression of target genes for IFN and NF-κB signaling pathways. CXCR2 is a chemokine receptor expressed in different types of cells including leukocytes (Cheng et al., 2019), tumor cells (Acosta and Gil, 2009), endothelial cells (Li et al., 2018), chondrocytes (Sherwood et al., 2015), and mesenchymal cells (Kwon et al., 2021). Ligands of CXCR2 include CXCL family chemokines and could activate typical G-protein-mediated signaling cascades that regulate a wide range of cellular functions (Cheng et al., 2019). The level of CXCL8, the most well-known ligand for CXCR2, is increased in human fracture callus (Hoff et al., 2016; Edderkaoui, 2017), while CCL and CXCL family chemokines are among the most upregulated genes in aged fracture callus (Hebb et al., 2018). However, we did not detect the expression of putative CXCR2 ligands in our dataset. This observation is not surprising as chemokines are typically secreted by endothelial and immune cells (Turner et al., 2014), which were excluded from our study. Nevertheless, we identified *Annexin A1* (Supplementary Figure S7D) and *Cathelicidin* (Supplementary Figure S8B) as specific incoming signals (ligands) for cluster 1.2. Both ligands have been shown to be antagonists for CXCR2 (Zhang et al., 2009; Drechsler et al., 2015). Thus, it is likely that CXCR2 signaling is activated by chemokines produced by immune cells in the fracture callus micro-environment, leading to an increased inflammatory osteogenic population, while callus SSPCs produce negative regulative ligands to inhibit inflammation.

CXCR2 promotes inflammation (Bartek et al., 2008) and promotes cellular senescence *via* Rb/p16^INK4a^ and p53/p21^WAF1/CIP1^ signaling pathways (Acosta et al., 2008; Acosta and Gil, 2009). We did not detect a significant difference in the expression levels of senescent genes (p16 ^INK4a^, p21^WAF1/CIP1^, and p18^INK4c^) between house-keeping and inflammatory osteogenic populations nor the expression of SASP factors such as *Tgfb1* (Supplementary Figure S9). Thus, it is unlikely that the CXCR2^high^ cells in the inflammatory osteogenic population are senescent cells.

We discovered the inflammatory osteogenic cluster is under the control of IRF and NF-κB family transcription factors. IRF and NF-κB family transcription factors initiate the transcription of interferon (IFN) and other inflammatory cytokines (Yanai et al., 2012; Zhao et al., 2015), substantiating the inflammatory phenotype of this subset. Being an important cytokine, the role of IFN in osteoimmunology has been thoroughly investigated (Tang et al., 2018), while less is known about its role in SSPCs in fracture repair. Low levels of IFN have been reported in osteoblasts (Ruiz et al., 2007) and mesenchymal stem cells (Duque et al., 2009) and have been shown to promote osteoblastic and inhibit adipogenic differentiation (Duque et al., 2009). In addition, IFN-primed mesenchymal cells are immunosuppressive by impacting immune cells in the callus (Kim et al., 2018) and have shown promising results in clinical trials in non-union fractures (Medhat et al., 2019).

It is worth noting that the expression of *Irf* or *Nfkb* family genes in our dataset was low and not differentially expressed between clusters 1.1 and 1.2. Although this does not necessarily contradict our findings that IRFs are the master regulators for cluster 1.2, the expression level of a transcription factor does not always positively correlate with its response genes (Joshi et al., 2012). For example, the copy number of altered transcription factors may also affect the expression level of downstream target genes. These altered transcription factors may still lead to the increased expression of target genes but could not be recognized in RNA sequencing (Joshi et al., 2012). Another factor that may affect the expression level of a transcription factor is whether it is a target gene of itself as in a positive regulation.

Currently, we do not know the exact role of the inflammatory osteogenic sub-cluster in fracture healing apart from that they have decreased osteogenic capacity. Since fracture healing is impaired in aged mice, which is accompanied by increased inflammatory osteogenic cells, we suspect they have a detrimental effect on healing. Thus, the interaction between the osteogenic-inflammatory cluster with other SSPC clusters as well as non-SSPC callus cells should be further investigated. Our initial ligand–receptor analysis with Cellchat (Jin et al., 2021) and NicheNet (Browaeys et al., 2020) did not detect strong outgoing signals between inflammatory osteogenic cells and other SSPC clusters (Supplementary Figure S7), which implies this cell population may mainly affect CD45^+^ myeloid lineage cells that are not included in our samples. The impact and mechanisms of the inflammatory osteogenic population on aging fracture repair warrant further investigation.

In summary, the osteogenic SSPC population can be further sub-clustered into house-keeping and inflammatory populations, in which the former is decreased and the latter, with decreased osteogenic capacity, is markedly increased in aged callus. The combination of housekeeping function stimulation and IRF and NF-κB inhibition may represent a new therapy for fracture in aging.

## Data Availability

The datasets presented in this study can be found in online repositories. The names of the repository/repositories and accession number(s) can be found below: GEO, GSE199755.
