# Supplementary material for: Aged Callus Skeletal Stem/Progenitor Cells Contain an Inflammatory Osteogenic Population With Increased IRF and NF-κB Pathways and Reduced Osteogenic Potential
Source: Front Mol Biosci. 2022 Jun 9;9:806528. doi: 10.3389/fmolb.2022.806528 (PMC9218815; doi:10.3389/fmolb.2022.806528)
Supplement: Supplementary file 1 [file Image2.pdf]

Supplementary Figure 4

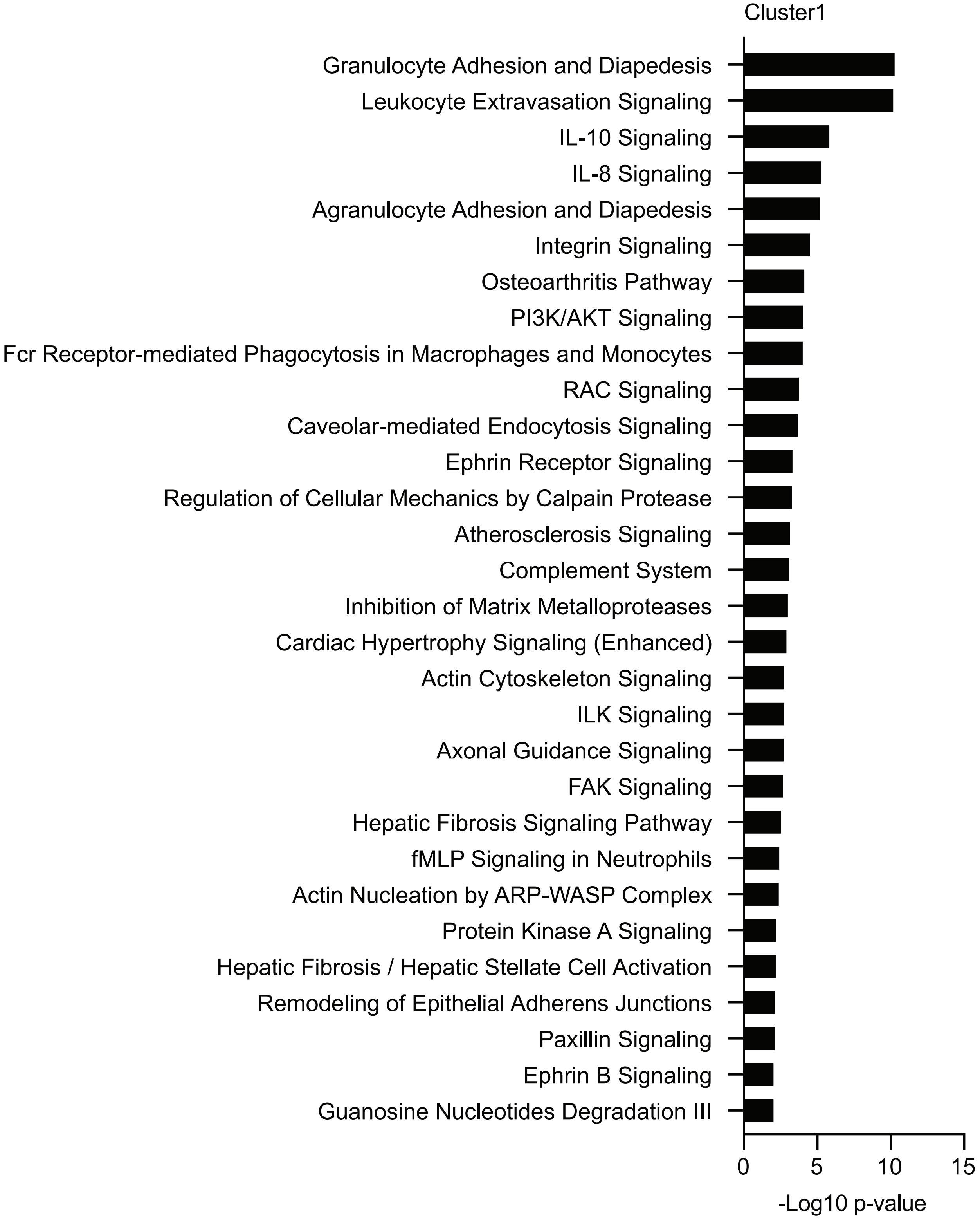

Supplemental Figure 4. Top 30 pathways in the osteogenic cluster. IPA analysis using 279 DEGs with Log2Fold changes greater than 0.5 and the top 30 upregulated pathways and their -Log10p-values were shown.

Supplementary Figure 5

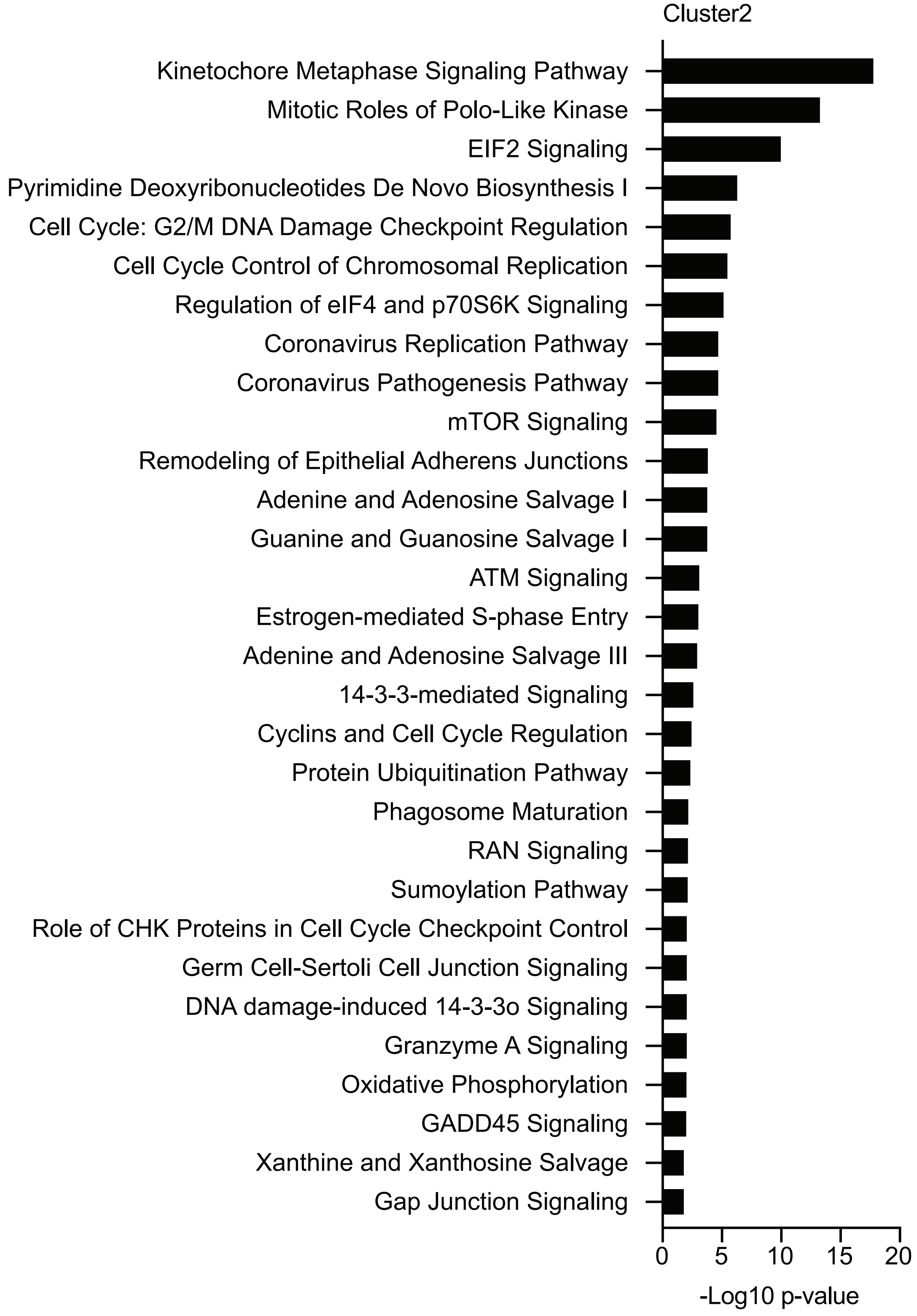

Supplemental Figure 5. Top 30 pathways in the proliferating cluster. IPA analysis using 685 DEGs with Log2Fold changes greater than 0.5 and the top 30 upregulated pathways and their -Log10p-values were shown.

Supplementary Figure 6

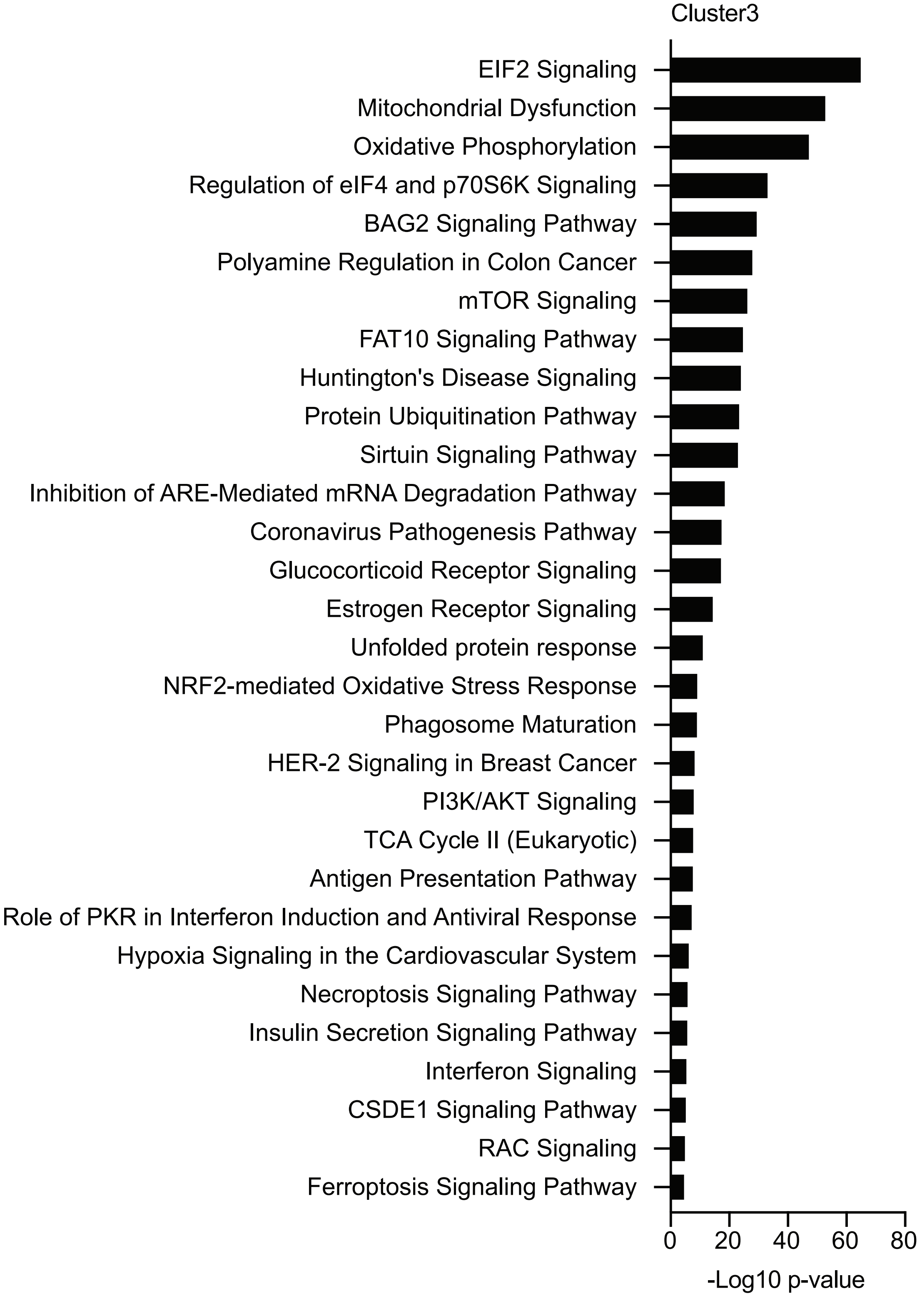

Supplemental Figure 6. Top 30 pathways in the adipogenic cluster. IPA analysis using 1715 DEGs with Log2Fold changes greater than 0.5 and the top 30 upregulated pathways and their -Log10p-values were shown.

Supplementary Figure 7

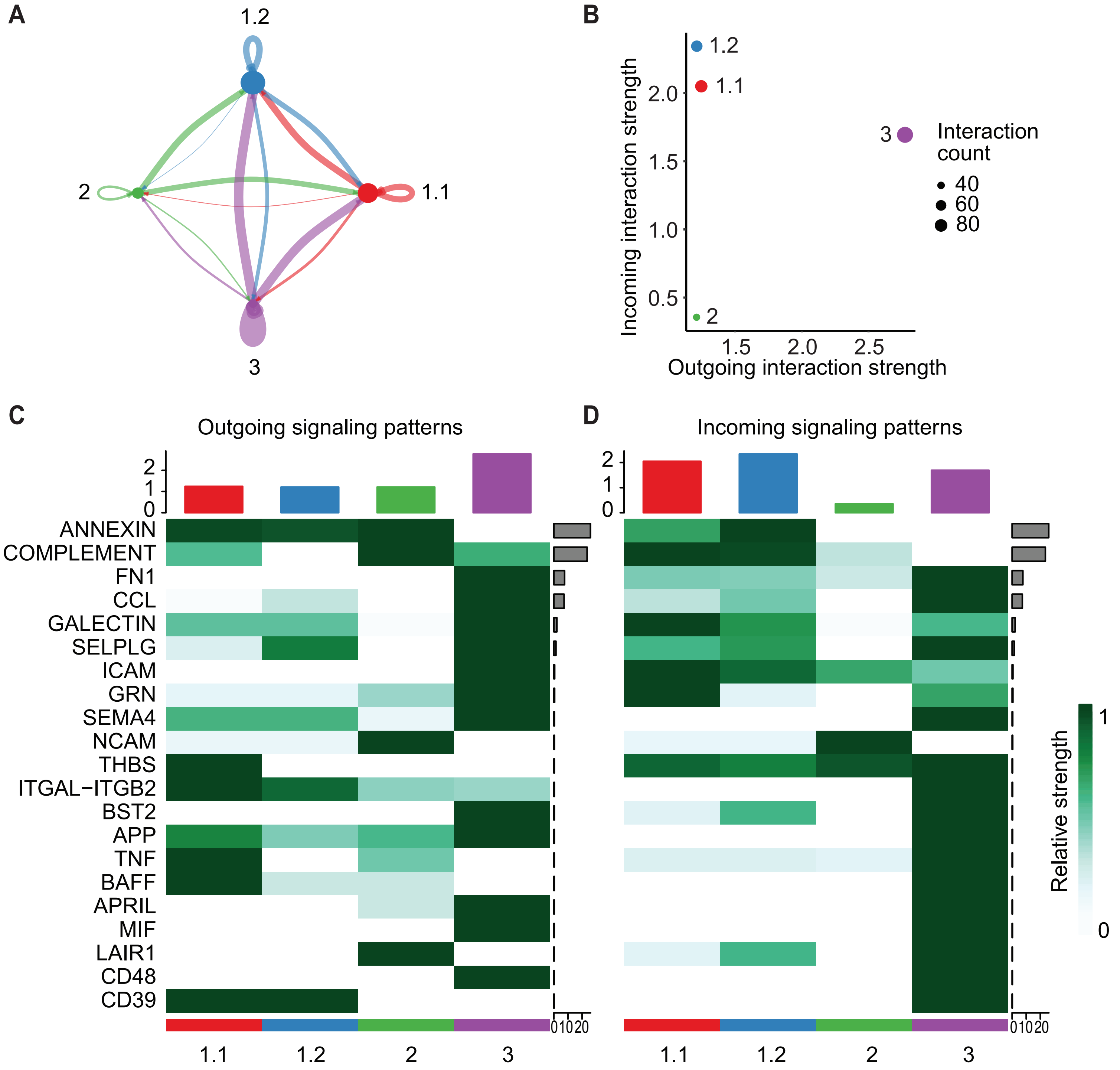

Supplemental Figure 7. Adipogenic cluster produces the most outgoing signals (ligands) for house-keeping and inflammatory osteogenic clusters. Ligand-receptor interaction using CellChat/R between SSPC sub-clusters. (A) Circle plot showing interaction strength between SSPC sub-clusters. Arrows and line color indicate direction (ligand —receptor) and line thickness indicates the sum of discovered ligand-receptor pair and strength between clusters. (B) Comparison of total outgoing (ligand) and incoming (receptor) interaction strength of the 4 sub-clusters indicating that cluster 3 adipogenic cells produce the most ligands, while cells in cluster 1.1 house-keeping and cluster 1.2 inflammatory osteogenic populations received the most signals. (C-D) Heatmap showed the relative importance of the outgoing (ligand) and incoming (receptor) interaction between sub-clusters.

Supplementary Figure 8

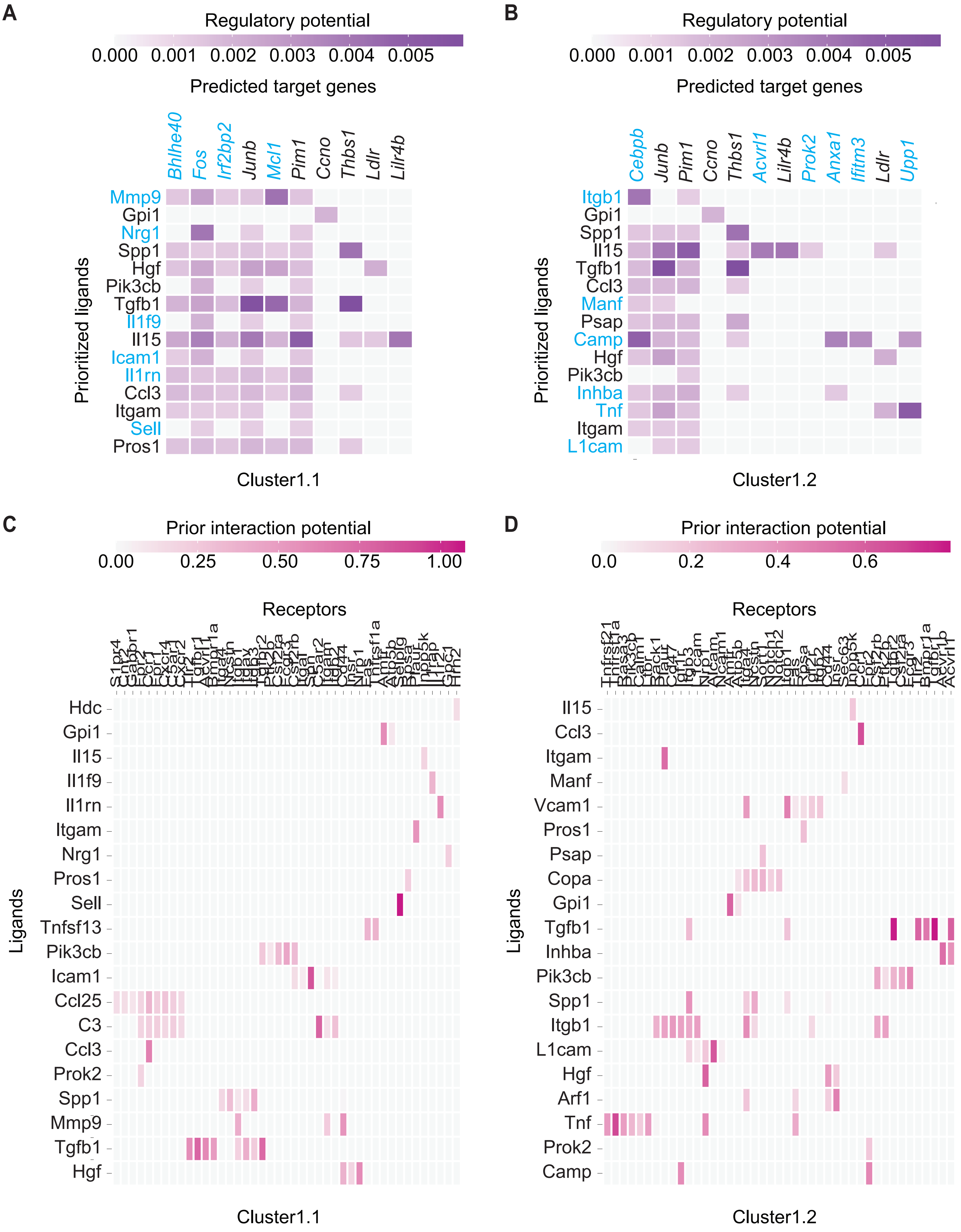

Supplementary Figure 9

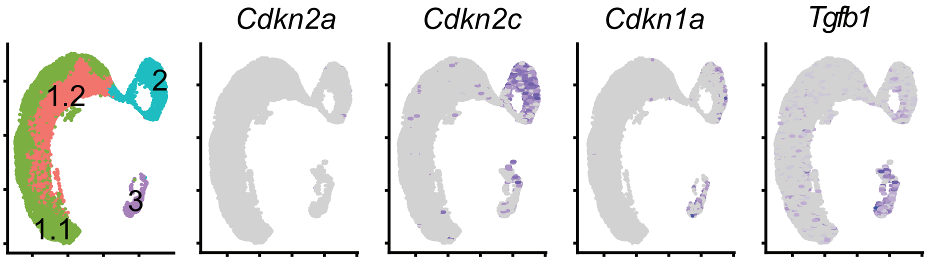

Supplemental Figure 9. Senescent cell markers are predominantly expressed in the proliferating and adipogenic clusters. UMAP shows the expressing of senescent cell marker genes Cdkn2a (p16), Cdkn2c (p18), Cdkn1a (p21), and senescence-associated secreted factors Tgf[illegible]1 are predominantly in cluster 2 proliferating and cluster 3 adipogenic cells.

Supplementary Table 1

Runx2	Forward	5’-CCTGAACTCTGCACCAAGTC-3’
	Reverse	5’-GAGGTGGCAGTGTCATCATC-3’
Acta2	Forward	5’-GTCCCAGACATCAGGGAGTAA-3’
	Reverse	5’-TCGGATACTTCAGCGTCAGGA-3’
Col1a1	Forward	5’-GCTCCTCTTAGGGGCCACT-3’
	Reverse	5’-CCACGTCTCACCATTGGGG-3’
Alp	Forward	5’-ACAGCAAGCCCAAGAGACCTTGAA-3’
	Reverse	5’-TGGTGTAGCCTGGCCCTTAAGGATT-3’
Sox9	Forward	5’-CGGAACAGACTCACATCTCTCC-3’
	Reverse	5’-GCTTGCACGTCGGTTTTGG-3’
Col10a1	Forward	5’-CTTTGTGTGCCTTTCAATCG-3’
	Reverse	5’-GTGAGGTACAGCCTACCAGTTTT-3’
Mki67	Forward	5’-AGCACAAAGAGACGGTCTAAGA-3’
	Reverse	5’-CTCTGCCTCGTGACTGTGTT-3’
Hist1h1b	Forward	5’-CTCCTGTAGAGAAGTCTCCCG-3’
	Reverse	5’-GCAGAAACAGCCTTAGTGATGAG-3’
Lpl	Forward	5’-GGGAGTTTGGCTCCAGAGTTT-3’
	Reverse	5’-TGTGTCTTCAGGGGTCCTTAG-3’
Apoe	Forward	5’-CTGACAGGATGCCTAGCCG-3’
	Reverse	5’-CGCAGGTAATCCCAGAAGC-3’
Cxcr2	Forward	5’-ATGCCCTCTATTCTGCCAGAT-3’
	Reverse	5’-GTGCTCCGGTTGTATAAGATGAC-3’
Ifitm1	Forward	5’-GACAGCCACCACAATCAACAT-3’
	Reverse	5’-CCCAGGCAGCAGAAGTTCAT-3’
S100a6	Forward	5’-CATATGCATGCCCTCTG-3’
	Reverse	5’-CGGATCCTTATTTCAGAGCT-3’
Slpi	Forward	5’-GGCCTTTTACCTTTCACGGTG-3’
	Reverse	5’-TACGGCATTGTGGCTTCTCAA-3’
Actin	Forward	5’-GTCAGGATCTTCATGAGGTAGT-3'
	Reverse	5‘-ACCCAGATCATGTTTGAGAC-3'

Supplemental Table 1. List of primers for qPCR analysis.

Supplementary Table 2

Gene	Protein
Acta2	Actin Alpha 2, Smooth Muscle
Acvrl1	Activin A receptor like type 1
Adgre1	F4/80
Anxa1	Annexin A1
Apoe	Apolipoprotein E
Camp	Cathelicidin Antimicrobial Peptide
Cd14	Monocyte Differentiation Antigen CD14
Cd164	CD164
Cd19	B-Lymphocyte Surface Antigen B4
Cd3g	T-Cell Surface Glycoprotein CD3 Gamma Chain
Cd79a	B-Cell Antigen Receptor Complex-Associated Protein Alpha Chain
Cd79b	B-Cell Antigen Receptor Complex-Associated Protein Beta Chain
Cebpb	Interleukin 6-Dependent DNA-Binding Protein
Col1a1	Collagen type I alpha 1 chain
Col2a1	Collagen type II alpha 1 chain
Col5a1	Collagen type V alpha 1 chain
Crip1	Cysteine-Rich Protein 1
Csf1r	Colony Stimulating Factor 1 Receptor
Cxcl8	Interleukin 8
Cxcr2	C-X-C Chemokine Receptor Type 2/Interleukine 8 Receptor Type B
Fcgr3b	CD16b
Fn1	Fibronectin 1
Fut4	CD15
Hist1h1b	Histone Cluster1 H1 Family Member B
Hist1h2ap	Histone Cluster1 H2A Family Member P
Ifitm1	Interferon Induced Transmembrane Protein 1
Ighg1	Immunoglobulin Heavy Chain of IgG
IRF1~9	Interferon Regulatory Factor 1~9
Irf2bp2	Interferon Regulatory Factor 2 Binding Protein 2
Lpl	Phospholipase A1
Ly6a	SCA-1
Ly6e	SCA-2
Ly76	TER-119
Mcam	CD146
Mki67	Marker of Proliferation Ki-67
Mmp8	Matrix Metalloproteinase 8
Mmp9	Matrix Metalloproteinase 9
Nt5e	CD73
Pdpn	Podoplanin
Pecam1	CD31
Ptprc	CD45
Retnlg	Resistin like gamma
Runx2	RUNX family transcription factor 2
S100a4	S100 Calcium Binding Protein A4
S100a6	S100 Calcium Binding Protein A6
Slpi	Secretory Leukocyte Peptidase Inhibitor
Sox9	SRY-Box Transcription Factor 9
Stat1/2	Signal Transducer And Activator of Transcription 1/2
Thbs1	Thrombospondin 1
Top2a	DNA Topoisomerase II Alpha
Tpsab1	Tryptase Alpha/Beta-1
Trac	T Cell Receptor Alpha Constant
Tuba1b	Tubulin Alpha 1b

Supplementary Table 2 (Continued)

Gene	Protein
Upp1	Uridine Phosphorylase 1
Vwf	Vone Willebrand Factor
Zmpste24	Zinc metallopeptidase STE24

Supplemental Table 2. List of genes and corresponding proteins in the manuscript.
