# Supplementary material for: Aged Callus Skeletal Stem/Progenitor Cells Contain an Inflammatory Osteogenic Population With Increased IRF and NF-κB Pathways and Reduced Osteogenic Potential
Source: Front Mol Biosci. 2022 Jun 9;9:806528. doi: 10.3389/fmolb.2022.806528 (PMC9218815; doi:10.3389/fmolb.2022.806528)
Supplement: Supplementary file 2 [file Image1.pdf]

Supplementary Figure 1

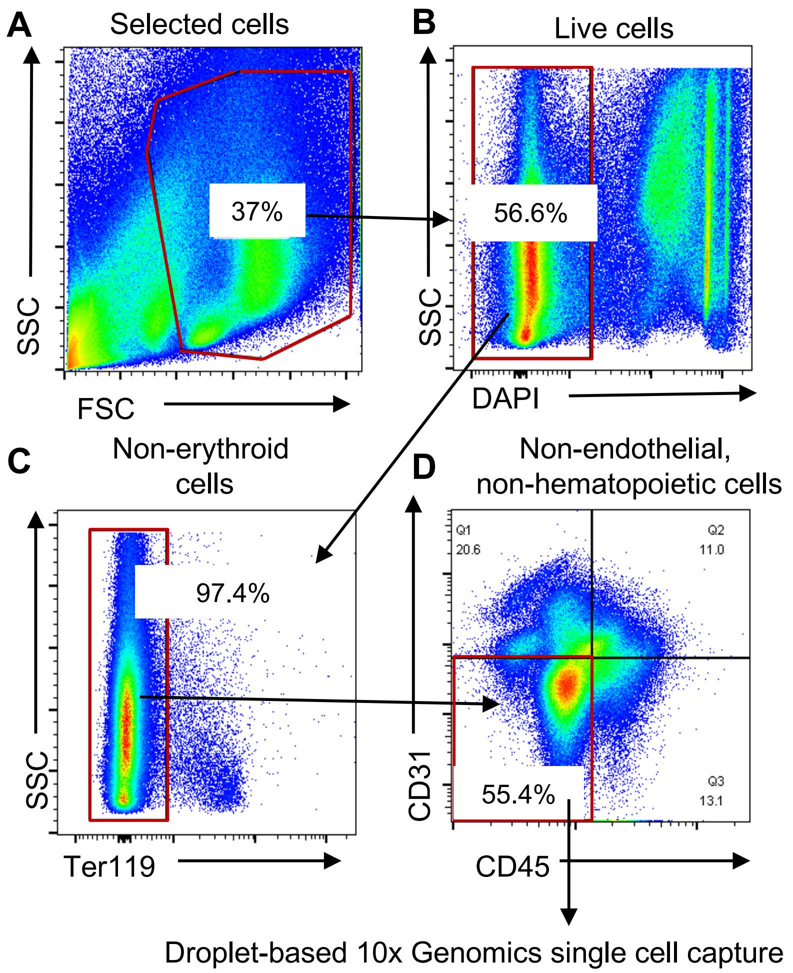

Supplemental Figure 1. FACS gating strategy for the isolation of mouse non-endothelia, non-hematopoietic SSPC cells. Representative FACS profile for young (4-month-old) and aged (21-month-old) mice are shown after the day 10 post fracture, which were sequentially sorted to exclude debris (A), and enrich for live (DAPI-, B), non-blood cells (Ter119-, C) and non-endothelial, non-hematopoietic cells (CD45-CD31-, D).

Supplementary Figure 2

A

Cell type	Gene	Protein	Total count
B cell	*Ms4a1*	CD20	140.85
	Cd19	CD19	76.60
T cell	*Cd3g*	CD3	16.01
	Trac	T cell receptor alpha constant	3.09
Plasma cell	*Ighg1*	Immunoglobulin heavy constant gamma1	70.05
Monocyte	*Cd14*	CD14	305.94
Macrophage	*Adgre1*	F4/80	450.93
	Csf1r	CSF1R	1409.31
Neutrophil	*Fcgr3b*	CD16	undetected
	Fut4	CD15	410.15
Mast cell	*Tpsab1*	Tryptase alpha/beta 1	undetected
Fibroblast	*Thy1*	Thy-1 Membrane Glycoprotein	11.12
	Pdpn	Podoplanin	172.94
Endothelial	*Emcn*	Endomucin	undetected
	Vwf	von Willebrand factor	5.85
Epithelial	*Krt6a*	Keratin 6A	undetected

B

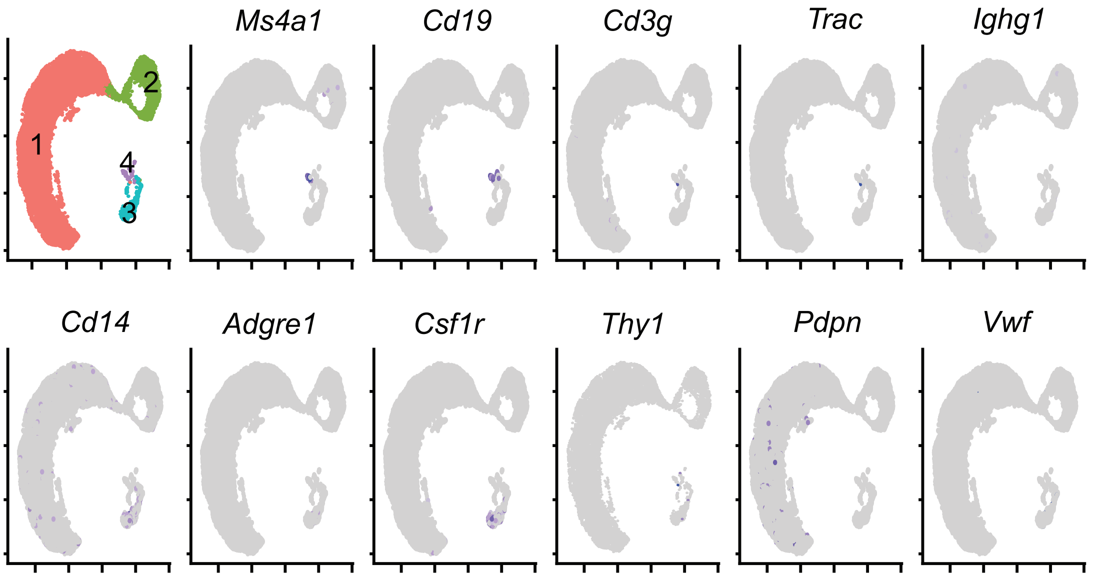

Supplemental Figure 2. Expression of putative markers for CD45+CD31+ cell types were low in callus SSPCs. (A) Table show putative markers genes and their corresponding proteins for CD45+ hematopoietic lineage cells, including B cell, T cell, plasma cell, monocyte, macrophage, neutrophil, and mast cells, and for fibroblast, endothelial cells, and epithelial cells. (B) Distribution of detected putative marker genes on UMAP.

Supplementary Figure 3

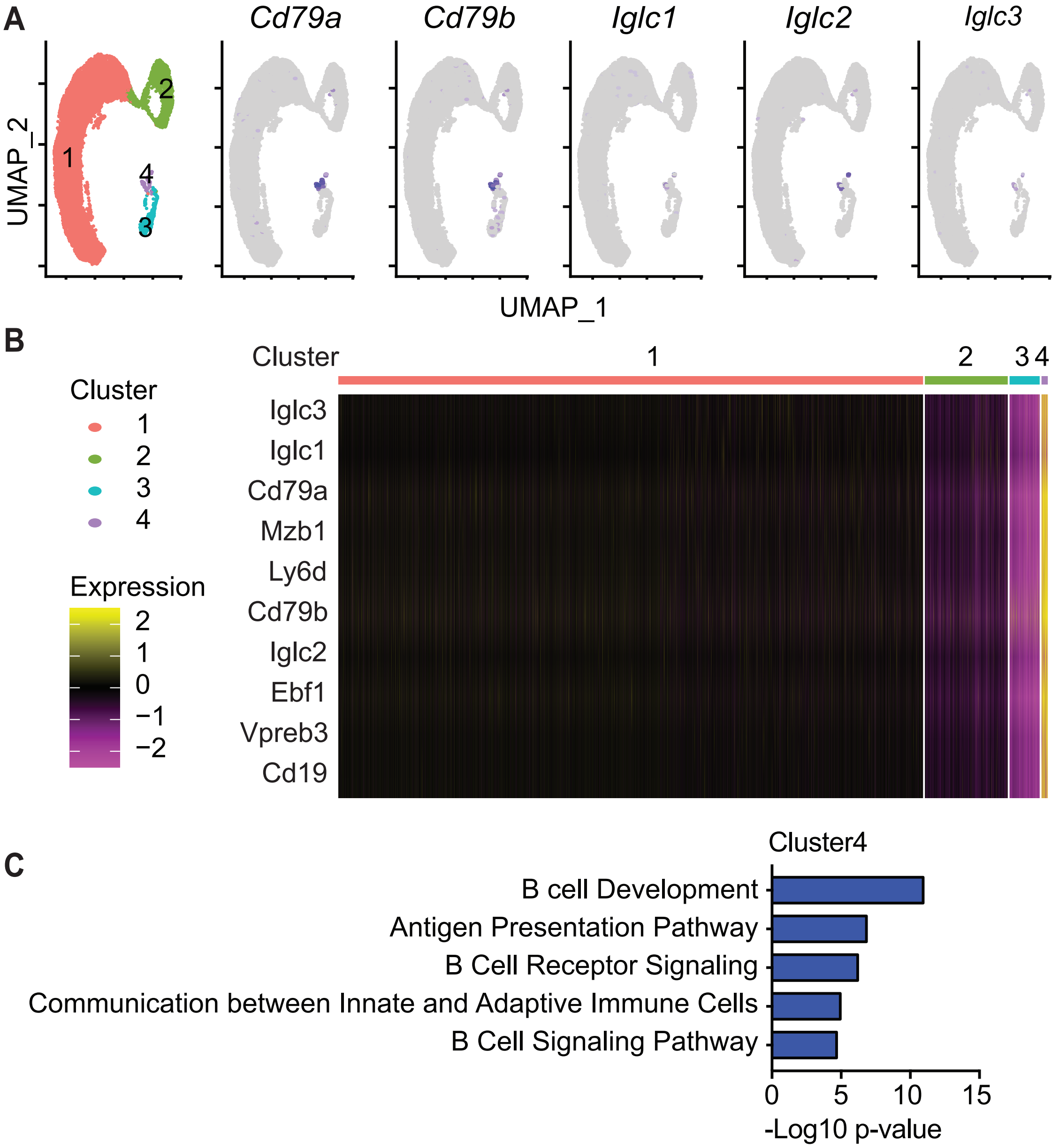

Supplemental Figure 3. Cluster 4 cells are enriched B cell-related genes and upregulated pathways. Cluster 4 (124 cells) were used for bioinformatics analysis. (A) B cell marker genes (CD19, CD79, CD20) and B cell function genes (Immunoglobin lambda constant) were differentially expressed in cluster 4. (B) Heatmap showing the top 10 DEGs of cluster 4 were related to B cells. (C) IPA analysis using the DEGs with Log2Fold changes greater than 0.5 and the top 5 upregulated pathways were all related to B cell function.
